# Full-length transcriptome of *Camellia perpetua* reveals candidate *SCPL*1*A* gene family members involved in galloylated catechins biosynthesis

**DOI:** 10.5511/plantbiotechnology.25.0317a

**Published:** 2025-12-25

**Authors:** Yongbiao Deng, Bo Wang, Jingjian Li, Chao Xiong, Baojiao Huang, Lisheng Wang, Bo Zhao

**Affiliations:** 1 Guangxi Key Laboratory of Drug Discovery and Optimization, School of Pharmacy, Guilin Medical University, Guilin 541199, China; 2 Hubei Institute for Drug Control, Wuhan 430075, China; 3 NMPA Key Laboratory of Quality Control of Chinese Medicine (HuBei), Wuhan 430075, China; 4 School of Life Science and Technology, Wuhan Polytechnic University, Wuhan 430023, China

**Keywords:** *Camellia perpetua*, correlation analysis, full-length transcriptome, galloylated catechins biosynthesis, RT-qPCR analysis, *SCPL*1*A* gene family

## Abstract

Catechins includ galloylated catechins and non-galloylated catechins, among which galloylated catechins exhibit stronger antioxidant, anti-inflammatory and anti-cancer activities. Section *Chrysantha* Chang, the only group of yellow *Camellia* with rich catechins in their flowers, is a common health drink in southern China. To date, few studies have examined galloylated catechins biosynthesis in flowers of this group. To enrich the genetic information of the galloylated catechins biosynthesis, the ONT sequencing platform was used to perform full-length transcriptome sequencing of *C. perpetua* flowers and 7,972,574 transcripts was identified, including 42,883 simple sequence repeats (SSRs), 41,961 coding sequences (CDSs) and 2,602 long non-coding RNAs (lncRNAs). 36,516 transcripts were successfully annotated, and 147 critical enzyme-encoding genes were identified as involved in the galloylated catechins biosynthesis pathway, including 17 *CpSCPL*1*A* genes. Bioinformatics analysis revealed that each *CpSCPL*1*A* protein consisted of 427–506 amino acids, and all *CpSCPL*1*A* proteins were divided into 5 groups with conserved motifs 1, 4, 5, 6 and 8. Based on the correlation analysis between the gene expression of 17 *CpSCPL*1*A* genes and the content of galloylated catechins, 11 candidate *CpSCPL*1*A* genes were identified to be involved in the biosynthesis of 4 types of galloylated catechins in *C. perpetua* flowers. The results enrich the transcriptome data for *C. perpetua* and provide valuable insights into the importance of the *CpSCPL*1*A* gene family members in the galloylated catechins biosynthesis.

## Introduction

The Sect. *Chrysantha* Chang belongs to the *Camellia* and is distinct for its unique yellow flowers (Jiang et al. 2023). Predominantly found in Guangxi and Yunnan, China and parts of Vietnam, this rare and endangered golden-yellow *Camellia* has been termed “Giant Panda of the Plant Kingdom” and the “Queen of *Camellia*” (Wu et al. 2020). *C. perpetua*, a species of the Sect. *Chrysantha* Chang is only distributed in Chongzuo of Guangxi, China and adjacent northern Vietnam. It is the only yellow *Camellia* that blossoms year-round, thereby captivating the interest of the horticultural community (Yu et al. 2023). Flowers of *C. perpetua* with abundant flavonoids compounds, exhibit anti-aging, antihypertensive, hypolipidemic, and anti-allergic effects and are routinely utilized to treat hypertension, malignant tumors, nephritis, dysentery, sores and irregular menstruation (Li et al. 2020; Qi 2016).

Flavonoids originate from the phenylpropanoid pathway, with the catechins biosynthesis pathway as a branch of this pathway (Supplementary Figure S1). Catechins serve as the terminal product of the flavonoid biosynthesis pathway in *Camellia* (Rani et al. 2012; Xiong et al. 2013). Clinical studies have demonstrated the myriad benefits of catechins for human health, including antioxidant effects on cardiovascular wellness, inhibiting the growth of cancer cells, preventing blood clot formation and reducing platelet aggregation and lipid regulation (Johnson et al. 2012; Sinija and Mishra 2008). Catechins, a subgroup of flavan-3-ols, are typically classified into seven categories: (+)-catechins (C), (−)-epicatechin (EC), (+)-gallocatechin (GC), (−)-epigallocatechin (EGC), (−)-epicatechin-3-gallate (ECG), (−)-epigallocatechin-3-gallate (EGCG) and (−)-gallocatechin gallate (GCG) (Tang et al. 2009; Wei et al. 2018). Among them, non-galloylated catechins, including C, EC, GC and EGC, are synthesized under the regulation of enzymes such as chalcone synthase (CHS), chalcone isomerase (CHI), dihydroflavonol reductase (DFR), leucoanthocyanidin reductase (LAR) and anthocyanidin reductase (ANR) (Guo et al. 2023; Punyasiri et al. 2004; Xiong et al. 2013). Finally, non-galloylated catechins (C, EC, GC and EGC) are enzymatically converted into galloylated catechins (CG, ECG, EGCG, GCG) by type 1A serine carboxypeptidase-like acyltransferases (*SCPL*1*A*s), galloylated catechins exhibit enhanced biological activities compared to their non-galloylated counterparts, including antioxidant, anti-inflammatory and anti-cancer activities, and these attributes make them valuable for applications in the pharmaceutical, food and cosmetic industries (Baranwal et al. 2022; Huang F et al. 2022). However, the role of the *SCPL*1*A* gene family in galloylated catechins biosynthesis of yellow flowers of *C. perpetua* is still not well understood, although many flavonoids biosynthesis related genes have been reported in flowers of Sect. *Chrysantha* Chang (Peng et al. 2011).

The genome of *C. perpetua* remains unavailable, severely limiting the exploitation of gene families. Oxford Nanopore Technologies (ONT) sequencing is a versatile platform widely used for full-length transcriptome sequencing as well as whole-genome shotgun sequencing and other applications. Compared to PacBio-SMRT sequencing technology, ONT sequencing technology offers the advantage of generating exceptionally long read lengths (up to 2 M base pair), which enhances the detection of complex genomic features. Moreover, ONT sequencing eliminates the need for PCR amplification, minimizing the risk of amplification-induced errors. ONT sequencing has emerged as a powerful tool in the study of gene families, gene expression, metabolic pathways, and genetic diversity across a spectrum of medicinal plants (Jiang et al. 2023; Mestre-Tomás et al. 2023; Park et al. 2018; Petersen et al. 2019), including *Iris lactea* var. *chinensis* (Ni et al. 2021), *Platycodon grandiflorus* (Yu et al. 2021) and *Pogostemon cablin* (Chen et al. 2019).

In this study, we conducted ONT sequencing to obtain full-length transcriptome of *C. perpetua* flowers at four distinct developmental stages, subsequently screened and identified *CpSCPL*1*A* gene family Members to analyze their genetic information, conservative domain, evolutionary relationships, and correlation analysis between the gene expression and the content of galloylated-type catechins. Our research generated a valuable dataset of full-length transcriptome for *C. perpetua* flowers and explored the critical *CpSCPL*1*A* gene family involved in galloylated-type catechins biosynthesis, thereby contributing to a deeper comprehension of the complex mechanisms for the flavonoid biosynthesis pathways in the Sect. *Chrysantha* Chang.

## Materials and methods

### Plant materials and sequencing

**Figure 1:**
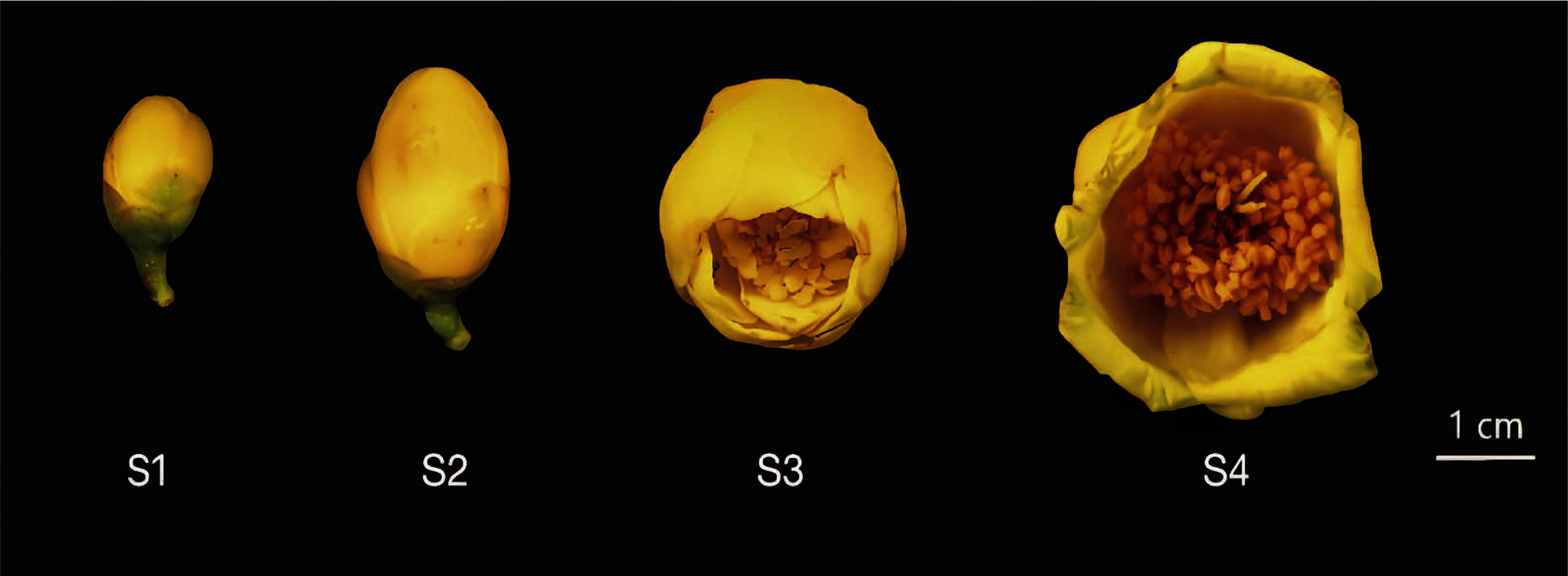
Figure 1. Flowers at four flowering stages: early buds (S1), yellowing flower buds (S2), semi-blooming flowers (S3) and full-bloomed flowers (S4).

The plants of *C. perpetua* were cultivated in a nursery at the Guangxi Institute of Botany, Chinese Academy of Sciences (Guangxi, China, E 110°18′54″, N 25°4′18″). Flowers at each of four developmental stages were collected with three biological replicates (from three similarly sized plants cultivated under identical conditions) on June 23, 2023 (Figure 1). The samples were rapidly frozen in liquid nitrogen and stored at −80°C for subsequent analysis.

Total RNA was extracted using the Total RNA Extractor Kit (Trizol) (B511311, Sangon Biotech Co., Ltd., Shanghai, China) following the manufacturer’s protocol after the collected flowers were ground and mixed in liquid nitrogen. The purity, concentration, and integrity of RNA were assessed using a Nanodrop (NanoDrop Technologies, Wilmington, USA) and an Agilent 2100 bioanalyzer (Agilent Technologies Inc, California, USA); only samples with an RNA Integrity Number (RIN)>8 were used in subsequent experiments. mRNA was enriched using oligo (dT) magnetic beads and reverse transcribed into cDNA with the SQK-PCS114 kit (Oxford Nanopore Technologies, Oxford, UK) for library construction and the MightyScript Plus First Strand cDNA Synthesis Master Mix (with gDNA diggalloylated) kit (B639252, Sangon Biotech Co., Ltd., Shanghai, China) for qRT-PCR. Sequencing was performed on the PromethION-48 system at Biomarker Technologies (Beijing, China) to obtain the raw sequencing data. The RNA-seq data were submitted to the NCBI Sequence Read Archive (SRA) database (Accession number: SRR30151566, SRR32367465 and SRR32367466).

### Transcriptomic analysis

Raw sequencing data were converted from fast5 to fastq format using GUPPY v4.2.2 (https://github.com/nanoporetech/minknow_api). Quality assessment and control were conducted with NanoComp and pychopper (https://github.com/nanoporetech/pychopper), Full-length reads were identified based on the presence of 5′ primers, 3′ primers, and poly-A tails, Chimeric reads were filtered out using NanoPack2, Error correction was performed, and the resulting high-quality reads were clustered to generate FLNC transcripts (De Coster and Rademakers 2023). These sequences are further processed and corrected using isONcorrect (Sahlin and Medvedev 2021). The processed data were then reconstructed using Lyric (https://github.com/guigolab/LyRic) to generate non-redundant transcripts. Protein-coding sequences were predicted using TransDecoder v5.7.1 (https://github.com/TransDecoder/TransDecoder) with default settings, where full-length transcripts longer than 100 amino acids were scored, and the highest-scoring ORF was selected to represent the coding sequence (CDS). Using BLAST, functional annotation of the non-redundant transcripts was performed by aligning them to several databases, including GO, KEGG, KOG, Pfam, SwissProt, COG, eggNOG and NR.

Simple sequence repeats (SSRs) were identified in all transcripts longer than 500 base pairs using MISA v1.0 with default parameters (Beier et al. 2017). Transcripts without protein-coding potential were identified as candidate lncRNAs using Pfam, CPAT v1.2 (https://rna-cpat.sourceforge.net/), CNCI v2.0 (https://github.com/www-bioinfo-org/CNCI) and CPC v1.0 (https://cpc.gao-lab.org/) (Kong et al. 2007). Finally, open reading frames (ORFs) in all candidate lncRNAs were predicted using EMBOSS getorf (Rice et al. 2000), and sequences over 100 base pairs were excluded from further analysis.

### Identification of *CpSCPL*1*A* gene family in *C. perpetua*

*SCPL*1*A* protein sequences of *C. sinensis* and *Arabidopsis thaliana* were downloaded from the Tea Plant Information Archive 2.0 (TPIA, http://tpia.teaplants.cn/index.html) and The Arabidopsis Information Resource (TAIR, https://www.arabidopsis.org/) (Supplementaty Table S1) (Berardini et al. 2015; Buchfink et al. 2015; Gao et al. 2024), respectively. A database was constructed using DIAMOND to search for potential *CpSCPL*1*A* protein sequences in *C. perpetua* transcripts (Buchfink et al. 2015). HMMER was utilized to download and build a hidden Markov model (HMM) with *SCPL*1*A* protein sequences from *C. sinensis* and *A. thaliana* as background files (Tang 2024); candidate *SCPL*1*A* protein sequences were identified by aligning these with *C. perpetua* transcripts (Potter et al. 2018). By integrating results from DIAMOND (Buchfink et al. 2015) and HMMER, *CpSCPL*1*A* proteins were identified. Additionally, GO annotations for *CpSCPL*1*A* proteins were retrieved using STRING (https://string-db.org/) (Szklarczyk et al. 2023). The gfanoo software was used to identify other key genes involved in the flavonoid synthesis pathway.

### Physicochemical properties and motif analysis of *CpSCPL*1*A* proteins

The Expasy tool (https://web.expasy.org/protparam/) was utilized to analyze the number of amino acids (aa), theoretical pI, instability index (II), and other physicochemical properties of the *CpSCPL*1*A* proteins (Huang et al. 2023). The NPSA server (https://npsa.lyon.inserm.fr/cgibin/npsa_automat.pl?page=/NPSA/npsa_sopma.html) was employed to determine the proportions of alpha helices, beta turns, extended strands and random coils in the *CpSCPL*1*A* proteins (Deléage 2017). The Plant-mPLoc tool (http://www.csbio.sjtu.edu.cn/bioinf/plant-multi/) was employed to predict the subcellular localization of *CpSCPL*1*A* proteins (Chou and Shen 2010). Additionally, the MEME suite (http://www.csbio.sjtu.edu.cn/bioinf/plant-multi/) identified motifs within the *CpSCPL*1*A* proteins, with parameters set to default except for the number of motifs, which was set to 10, Tbtools was used for motif visualization (Chen et al. 2020).

### Phylogenetic analysis of *CpSCPL*1*A* proteins

The *CpSCPL*1*A* protein sequences of *C. sinensis* were downloaded from the Tea Plant Information Archive 2.0 (TPIA, http://tpia.teaplants.cn/index.html) (Supplementary Table S2). The local pair algorithm in the MAFFT was employed to align these sequences (Rozewicki et al. 2019). The resulting alignments were trimmed using TrimAI software (Capella-Gutiérrez et al. 2009). A phylogenetic tree of *CpSCPL*1*A* proteins was constructed using the neighbor-joining (NJ) method in MEGA-X, with 1,000 bootstrap replicates (Kumar et al. 2018). Finally, iTOL (https://itol.embl.de/itol.cgi) was employed to visualize and annotate the phylogenetic tree (Letunic and Bork 2021).

### qRT-PCR analysis

The expression level of *CpSCPL*1*A* genes was detected via qRT-PCR using pre-synthesized cDNA as a template. qRT-PCR analysis was conducted using RotorGene Q (Qiagen, Beijing, China). Specific primers for *CpSCPL*1*A* genes were designed using Primer Premier 5.0 software (Supplementary Table S3), with the *18S* gene as the housekeeping gene for normalization. Each 20 µl reaction consisted of 10 µl of 2X SGExcel FastSYBR Mixture, 0.4 µl each of 0.4 µM forward and reverse primers, 2 µl of 50 ng cDNA and 7.2 µl of RNase-Free ddH2O. Amplification conditions were: initial denaturation at 95°C for 3 min, followed by 40 cycles of 95°C for 5 s and 60°C for 20 s. Fluorescence signals were collected in the FAM channel following the annealing/extension step, and melting curve analysis was performed at the end. Gene expression levels were quantified using the 2^−ΔΔCt^ method (Livak and Schmittgen 2001). Each experiment was conducted using three independent biological replicates and three technical replicates.

### Extraction and HPLC analysis of galloylated catechins

Galloylated catechins were quantified using high-performance liquid chromatography (HPLC). Flowers at various developmental stages were frozen and uniformly ground in liquid nitrogen. A 0.1 g sample (accurate to 0.0001 g) was precisely weighed and transferred to a 10 ml centrifuge tube. Subsequently, 10 ml of preheated (70°C) 70% methanol solution was added. The mixture was homogenized and incubated in a metal bath at 70°C for 10 min. After cooling to room temperature, the mixture was centrifuged at 3,500 rpm for 10 min, and the supernatant was collected. The residue was re-extracted using the same procedure, and the supernatants were combined and diluted to 10 ml with 70% methanol. The final solution was filtered through a 0.22 µm organic membrane and analyzed via HPLC. Galloylated catechins content was quantified using an Elite P1201 High-Performance Liquid Chromatograph (Elite Analytical Instrument Co., Ltd., Dalian, China) equipped with a reversed-phase C18 column (4.6 mm inner diameter, 250 mm length, 5 µm particle size) (Agilent Technologies, USA). UV detection was carried out at 278 nm, with the column temperature at 35°C. The injection volume was 10 µl, gradient elution at a 0.5 ml/min flow rate. Mobile phase A comprised 8% acetonitrile, 2% acetic acid and 90% pure water, while mobile phase B contained 90% acetonitrile, 8% pure water and 2% acetic acid. Standard solutions for EC, GCG, EGCG and ECG were prepared at 2.00 mg/ml concentrations (Shanghai Shifeng Biological Technology Co., Ltd, China). The HPLC chromatograms of the standard compounds and samples are shown in Supplementary Figure S2.

## Results

### Full-length transcriptome sequencing data output

After filtering out low-quality reads, Oxford Nanopore sequencing yielded 38.30 Gb of clean reads (Table 1). We obtained 7,972,574 full-length non-chimeric (FLNC) transcripts from these clean reads, representing 88.82% of the total. The average read length of transcripts, including 5′ primers, 3′ primers and poly (A) tails of FLNCs, was 1,163 base pairs (bp). Additionally, we acquired 90,245 consensus isoforms with an average length of 1,327 bp and an average quality score of 0.99, and the N50 length was 1,695 bp. Further sequence-structure analysis of the transcripts resulted in 57,487 unique transcripts with an average length of 1,385 bp. We also identified 41,961 coding sequences (CDSs), 42,883 simple sequence repeats (SSRs) and 2,602 long non-coding RNAs (lncRNAs) (Table 1).

**Table 1:** Table 1. Statistical data on the sequencing of full-length transcriptomes.

Data output	Number
Subread bases (Gb)	38.30
Subread number	9,833,772
Average subread length	1,385
Consensus N50	1,695
FLNC	7,972,574
Consensus number	90,245
MaxLength	9,574
Transcripts	57,487
SSR	42,883
LncRNA	2,602

### Functional annotation of transcripts and gene families related to galloylated catechins biosynthesis

**Figure 2:**
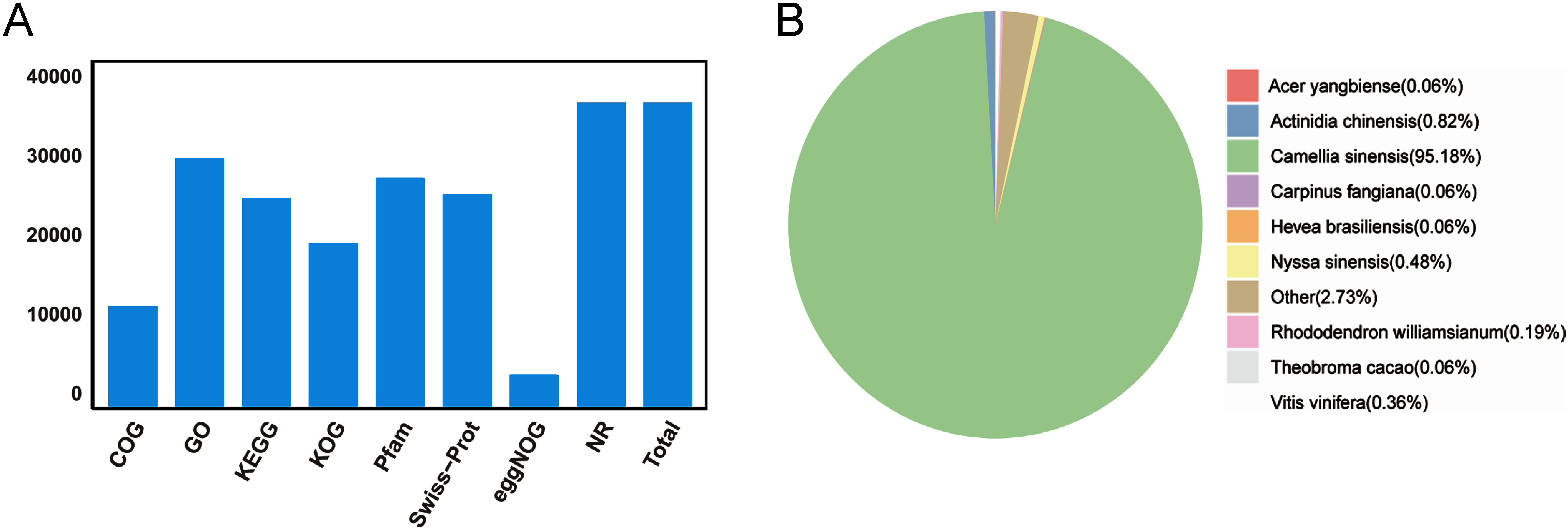
Figure 2. Annotated agave full-length genes in public databases (A) and species distribution based on Nr annotation (B).

**Figure 3:**
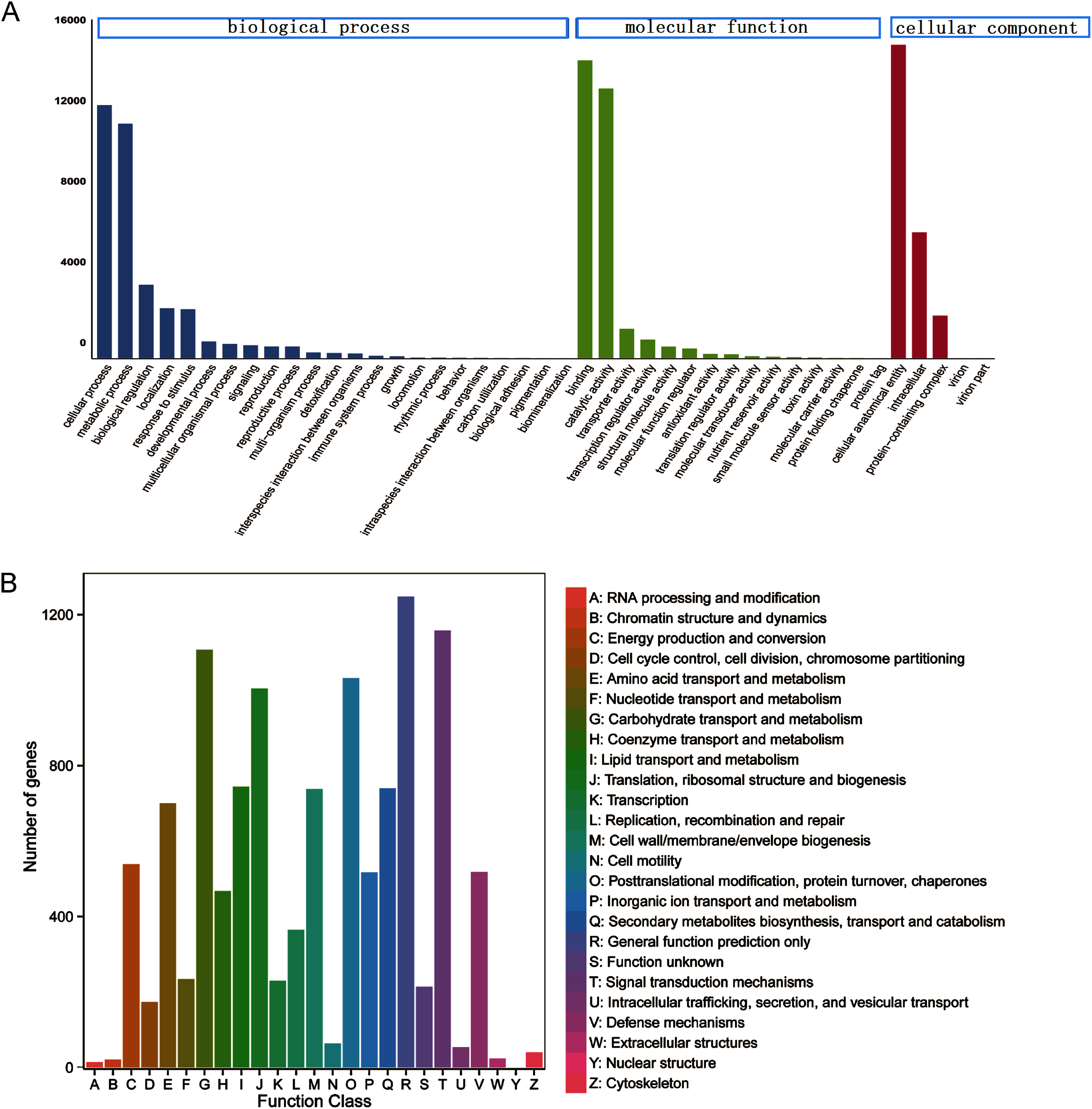
Figure 3. GO classifications of annotated agave full-length genes (A) and KOG classes of annotated agave full-length genes (B).

To obtain transcript annotation, BLAST was used to align transcripts against the NR, SwissProt, GO, COG, KOG, Pfam and KEGG databases. A total of 36,516 transcripts were successfully annotated, with 29,508, 24,593, 18,948, 2,717, 25,092, 10,753, 2,361 and 36,444 aligned to the GO, KEGG, KOG, Pfam, SwissProt, COG, eggNOG and NR databases, respectively (Figure 2, Supplementary Table S4). Notably, 97.74% of unigenes were annotated, demonstrating extensive functional coverage. Further analysis of the NR database revealed the closest phylogenetic relationship with *C. sinensis* (95.18%), indicating high accuracy in the transcriptome assembly and annotation of *C. perpetua*. Analysis of the GO and COG annotations (Figure 3) revealed that 11,624 transcripts were classified under the “metabolic process” category in GO. In comparison, 740 transcripts were assigned to the “Secondary metabolites biosynthesis, transport and catabolism” category in COG. A hidden Markov model (HMM) search was conducted on the aforementioned transcripts and encoded proteins, resulting in the identification of numerous genes implicated in the biosynthesis of galloylated catechins. These include LAR, DFR, F3H and *SCPL*1*A* (Supplementary Table S5).

### Identification and protein structure analysis of *CpSCPL*1*A* genes

We used DIAMOND and HMMER comparison tools to ensure accurate sequence extraction. Seventeen *CpSCPL*1*A* genes with complete coding sequences (CDS) and open reading frames (ORF) were identified in the full-length transcriptome of *C. perpetua* and were named *CpSCPL*1*A*1 to *CpSCPL*1*A*17 (Table 2). The analysis of the physicochemical properties revealed minimal variation in the protein sequences encoded by the *CpSCPL*1*A* gene family. Their protein lengths ranged from 427 to 506 amino acids, with theoretical pI values between 4.84 and 6.96, indicating their acidic nature. Instability indexes ranged from 33.70 to 45.90, with *CpSCPL*1*A*9 being the highest at 45.90 and *CpSCPL*1*A*6 being the lowest at 33.70, suggesting general stability in *Cp*SCPL1A proteins. Bioinformatic analysis of subcellular localization revealed that all proteins located in vacuoles, except for *CpSCPL*1*A*3 and *CpSCPL*1*A*7, which located outside the oxidoreductase. The secondary structure prediction for each member of *CpSCPL*1*A* gene family showed that the proportion of α-helix ranges from 28.78% to 33.72%, β-turns from 4.68% to 7.49%, and the remainder were composed of extended strands and random coils, indicating that the *CpSCPL*1*A* gene family exhibited a degree of conservation in their secondary structure. In addition, Using the gfnaoo software, we identified 13 4CL genes, 10 ANR genes, 5 CHS genes, 11 DFR genes, 11 PAL genes, and 1 UGT84 gene within the transcriptome dataset. These genes are known to play roles in the biosynthesis of diverse flavonoid compounds, including catechins.

**Table 2:** Table 2. Information and characteristics of the *CpSCPL*1*A* gene family in *C. perpetua* flowers.

Gene name	Peptide lengths	Isoelectric point	Instability index	Subcellular localization	Alpha helix%	Extended strand%	Beta turn%	Random coil%
*CpSCPL*1*A*1	480	6.83	39.18	Vacuole	31.04%	14.17%	6.46%	48.33%
*CpSCPL*1*A*2	500	6.06	39.06	Vacuole	33.60%	14.40%	6.40%	45.60%
*CpSCPL*1*A*3	496	5.34	33.76	Peroxisome	32.06%	14.31%	5.65%	47.98%
*CpSCPL*1*A*4	477	6.19	37.46	Vacuole	30.40%	14.88%	6.50%	48.22%
*CpSCPL*1*A*5	454	5.78	37.99	Vacuole	29.96%	15.20%	7.49%	47.36%
*CpSCPL*1*A*7	496	6.96	33.70	Vacuole	30.65%	13.71%	5.85%	49.80%
*CpSCPL*1*A*9	502	5.72	36.45	Peroxisome	32.67%	14.14%	4.98%	48.21%
*CpSCPL*1*A*10	446	6.31	40.74	Vacuole	30.94%	15.02%	6.95%	47.09%
*CpSCPL*1*A*12	463	4.84	45.90	Vacuole	29.37%	15.77%	6.26%	48.60%
*CpSCPL*1*A*13	506	5.43	42.89	Vacuole	34.78%	14.23%	5.53%	45.45%
*CpSCPL*1*A*14	470	5.27	40.97	Vacuole	29.57%	15.74%	7.23%	47.45%
*CpSCPL*1*A*15	483	5.26	40.38	Vacuole	28.78%	15.73%	6.21%	49.28%
*CpSCPL*1*A*16	480	5.03	38.35	Vacuole	30.83%	15.21%	6.25%	47.71%
*CpSCPL*1*A*17	427	5.32	39.70	Vacuole	33.72%	15.46%	4.68%	46.14%

### Motif analysis of *CpSCPL*1*A* proteins

**Figure 4:**
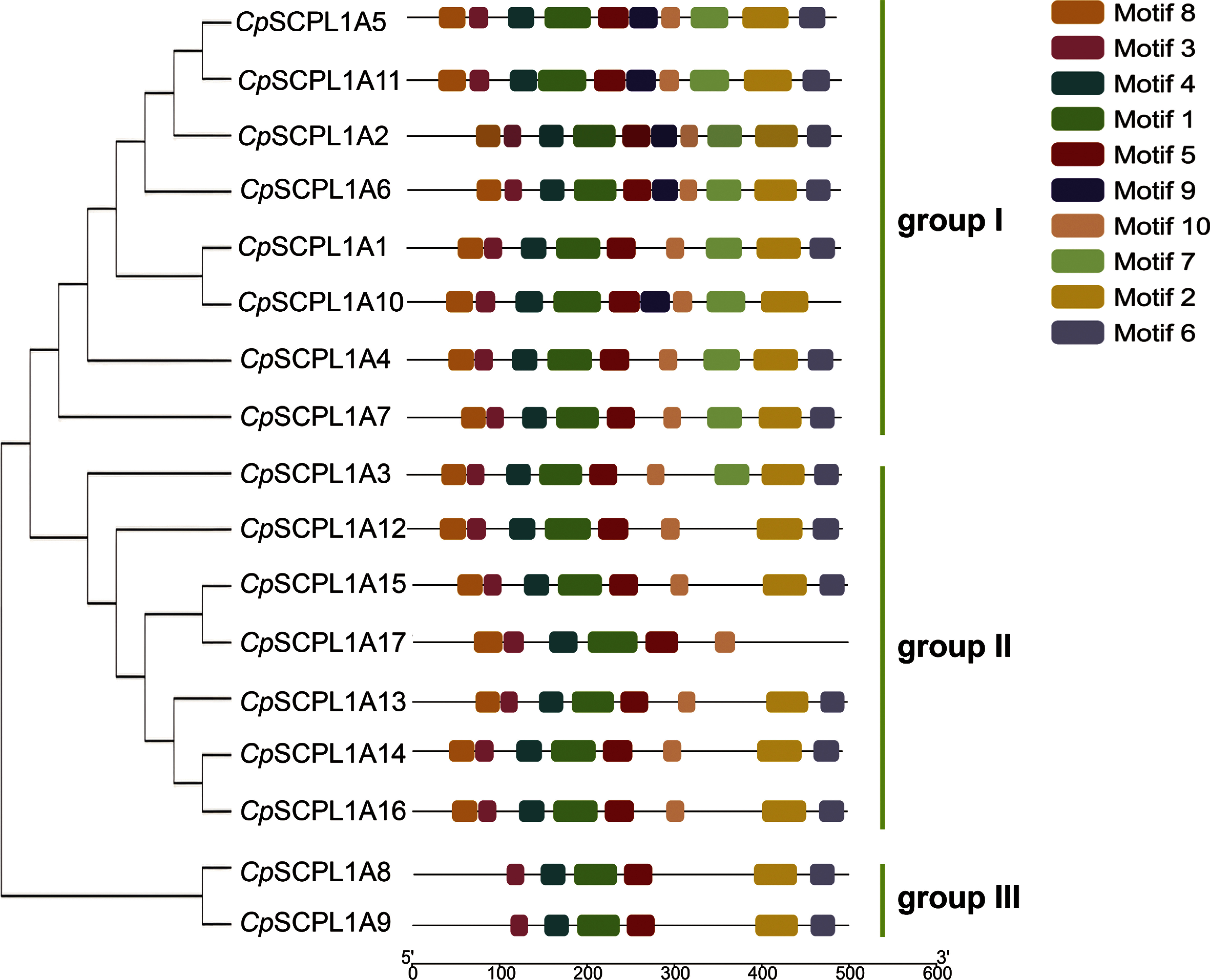
Figure 4. Analysis of phylogenetic relationships and conserved motifs of 17 *CpSCPL*1*A* proteins.

To explore the evolutionary relationships and conserved motif distribution within the *CpSCPL*1*A* proteins in *C. perpetua*, we employed the MEME-Suite to analyze the conserved domains of 17 *CpSCPL*1*A* proteins. Ten conserved motifs are named motif 1–10 (Figure 4, Supplementary Table S6). Motif1, motif4, motif5, motif6 and motif8 were notably present in all *CpSCPL*1*A* proteins. These motifs were contiguous in the sequence, located near the N-terminus, suggesting their crucial role in *CpSCPL*1*A* protein function. Additionally, motif3 was found in all *CpSCPL*1*A* proteins except *CpSCPL*1*A* 10 and *CpSCPL*1*A* 17, whereas motif9 was absent only in *CpSCPL*1*A* 8 and *CpSCPL*1*A* 9. Phylogenetic analysis revealed that certain motifs are unique to phylogenetic groups, for instance, motif7 was exclusively found in all group I members, whereas motif10 was unique to group II (Figure 4). Compared to groups I and II, group III members had fewer motifs, specifically lacking motif2, motif7, motif9 and motif10, suggesting they may have some distinct biological functions.

### Phylogenetic analysis of *CpSCPL*1*A* proteins

**Figure 5:**
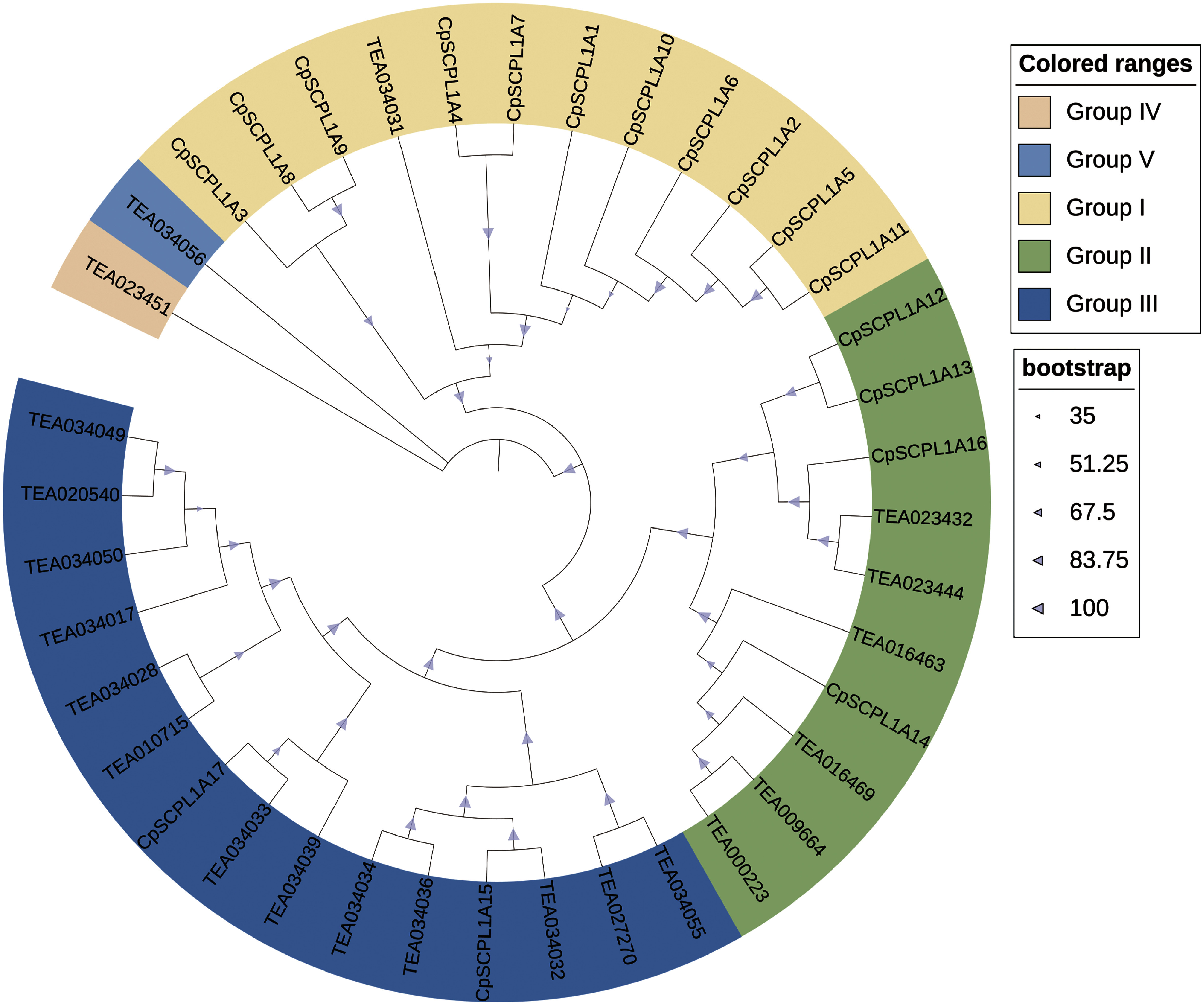
Figure 5. Phylogenetic analysis of *SCPL*1*A* protein in *C. sinensis* and *C. perpetua*. The figure shows that different colors represent different groups, and the triangle size represents different bootstrap values.

To elucidate the phylogenetic relationship within the *CpSCPL*1*A* proteins of *C. perpetua*, we constructed NJ tree including 17 *CpSCPL*1*A* proteins of *C. perpetua* and 22 *CsSCPL*1*A* proteins from *C. sinensis* (Supplementary Table S2). Phylogenetic analysis showed that all *SCPL*1*A* proteins could be categorized into Group I~V (Figure 5). Specifically, Group I comprised 11 *CpSCPL*1*A* proteins (*CpSCPL*1*A* 1–11) and 1 *CsSCPL*1*A* protein, while Group II included 4 *CpSCPL*1*A* proteins (*CpSCPL*1*A* 1–14, *CpSCPL*1*A* 16) and 6 *CsSCPL*1*A* proteins. Group III contains 2 *Cp*SCPL1A proteins (*CpSCPL*1*A* 15 and *CpSCPL*1*A* 17) and 13 *CsSCPL*1*A* proteins. Groups IV and V consist of TEA023451 and TEA034056, respectively. Notably, proteins within the same group exhibited high sequence homology, suggesting they may shared similar functions.

### The expression patterns of the *CpSCPL*1*A* gene at four flower development stages

**Figure 6:**
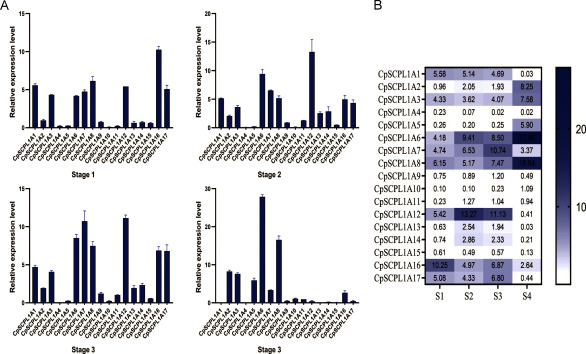
Figure 6. Relative expression levels of the *CpSCPL*1*A* gene at four flower development stages. Values represent the mean±SD, measured from at least three biological replicates. A: The relative expression of a single gene in different periods. B: Relative expression heat map of *CpSCPL*1*A* gene family at different developmental stages.

Expression patterns of 17 *CpSCPL*1*A* genes in *C. perpetua* flowers at four developmental stages were analyzed (Figure 1). The results showed that all genes were expressed with significant variations in expression levels across different stages (Figure 6A, B). Two genes (*CpSCPL*1*A*4, *CpSCPL*1*A*16) were highly expressed at the Stage 1 (S1), while *CpSCPL*1*A*11, *CpSCPL*1*A*12, *CpSCPL*1*A*13, *CpSCPL*1*A*14 were highly expressed the Stage 2 (S2). Notably, threel *CpSCPL*1*A* genes (*CpSCPL*1*A*7, *CpSCPL*1*A*9, *CpSCPL*1*A*17) were significantly expressed at the Stage 3 (S3), and *CpSCPL*1*A*2, *CpSCPL*1*A*3, *CpSCPL*1*A*5, *CpSCPL*1*A*6, *CpSCPL*1*A*8, *CpSCPL*1*A*10 were significantly expressed at the Stage 4 (S4).

### Combined analysis of RNAseq expression and metabolite data

**Figure 7:**
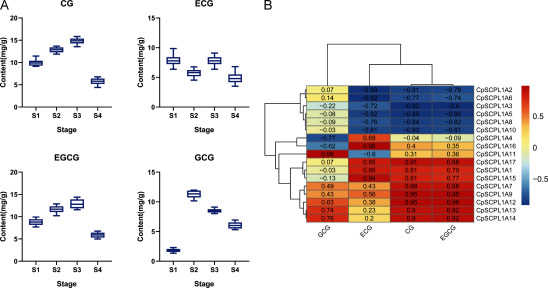
Figure 7. A: Relative content of 4 galloylated catechins at four flower development stages (The sample size of each stage is 25). B: Co-expression analysis of *SCPL*1*A* genes and 4 galloylated catechins. S1–S4, representing 4 stages of flower development.

We employed high-performance liquid chromatography (HPLC) to assess the contents of galloylated catechins across S1 to S4 (Figure 7A). The results showed that the contents of CG and EGCG initially increased, reached its peak at S3, and then decreased at S4. The content of ECG decreased at S2, increased at S3, and subsequently decreased at S4. The content of GCG increased from S1 to S2 but then continuously declined.

To investigate the role of the *CpSCPL*1*A* gene family in galloylated catechins biosynthesis, correlation analysis was performed between the quantitative fluorescence results of 17 *CpSCPL*1*A* genes and the content of galloylated catechins (Figure 7B). ECG content was positively correlated with 5 genes (*CpSCPL*1*A*7, *CpSCPL*1*A*9 and *CpSCPL*1*A*15–17), while negatively correlated with *CpSCPL*1*A*6. GCG content was positively associated with 5 genes (*CpSCPL*1*A*1, *CpSCPL*1*A*11–14), whereas showed a negative relation with 3 genes (*CpSCPL*1*A*3, *CpSCPL*1*A*8 and *CpSCPL*1*A*10). Interestingly, CG and EGCG content exhibited same regulatory pattern, with both being positively correlated with 8 genes (*CpSCPL*1*A*1, *CpSCPL*1*A*9, *CpSCPL*1*A*12–17) and negatively correlated with 6 genes (*CpSCPL*1*A*2, *CpSCPL*1*A*3, *CpSCPL*1*A*5, *CpSCPL*1*A*6, *CpSCPL*1*A*8, *CpSCPL*1*A*10). No significant correlations were observed for the remaining genes.

## Discussion

The transcriptomic profile serves as a vital bridge between the genomic blueprint and the biological activities governed by the proteome (Ayadi et al. 2018; Satam et al. 2023). Full length transcriptome sequencing, mainly through Oxford Nanopore Technology (ONT), compared to PacBio-SMRT sequencing technology, Oxford Nanopore Technologies (ONT) offers the advantage of generating exceptionally long read lengths, which enhances the detection of complex genomic features (Jain et al. 2016; Lu et al. 2016; Sigurpalsdottir et al. 2024). In this study, ONT sequencing was used to obtain the full-length transcriptome of *C. perpetua* flowers at four developmental stages, 38.30 Gb of clean data and a total of 7,972,574 full-length non-chimeric (FLNC) sequences were generated (Table 1). A comparative analysis with the full-length transcriptome of *C. nitidissima* petals using PacBio SMRT sequencing revealed that the number of unique transcripts obtained (45,372) was significantly lower than that (57,487) of *C. perceptua*. In *C. nitidissima*, 43,073 coding sequences (CDS) were identified, most ranging from 1,500 to 2,500 bp, in contrast, *C. perpetua* exhibited 41,961 CDS, primarily concentrated between 300 and 2,400 bp. This discrepancy may be attributed to differences in the sample material provided for sequencing.

After eliminating redundancies, we obtained 57,487 unique full-length transcripts (Table 1). Further analysis of Gene Ontology (GO) and Clusters of Orthologous Groups (COG) (Figure 3) annotations revealed a significant enrichment of genes associated with specialized metabolite biosynthesis. Delving deeper into the transcripts pertinent to these categories, we pinpointed 147 pivotal enzyme-encoding genes that contribute to the galloylated catechins biosynthesis pathway, including 17 *CpSCPL*1*A* genes termed *CpSCPL*1*A*1 to *CpSCPL*1*A*17 (Table 1). Most *CpSCPL*1*A* genes in *C. perpetua* exhibit analogous conserved motifs (Figure 4), such as motifs 1, 4, 5, 6 and 8 detected in all members, suggesting a highly conserved protein structure. Nonetheless, the precise functions of many of these conserved motifs remains unknown. A conserved motif of the plant gene family is a DNA sequence with specific functions highly preserved across various family members and intimately associated with gene expression regulation, protein structure, and function. The presence of a conserved motif may facilitate the RNA spliceosomes in recognizing splice sites, thus ensuring the accurate splicing of RNA (Liu et al. 2018).

Varying counts of *CpSCPL*1*A* genes have been identified in different plant species, such as 19 genes found in *Arabidopsis* (Swarbreck et al. 2008) and 16 in maize (Li et al. 2015) (Supplementary Table S1). The Whole Genome Duplication (WGD) event led to the widespread expansion of the *SCPL*1*A* gene family in the *C. sinensis*. Among the 22 *SCPL*1*A* gene families identified in the *C. sinensis* genome, 4 originated from WGD events that occurred approximately 30 to 40 million years ago. At the same time, 15 were generated through recent, species-specific tandem repeat amplification, a critical factor contributing to the diversity of the galloylated catechins biosynthetic pathway in *C. sinensis*. The phylogenetic analysis of the *SCPL*1*A* gene family revealed that *CpSCPL*1*A*12, *CpSCPL*1*A*13, and *CpSCPL*1*A*16 in *C. perpetua* were closely related to the highly expressed genes TEA023444 and TEA023432 in *C. sinensis* flowers (Figure 5). *CpSCPL*1*A*1, *CpSCPL*1*A*2, *CpSCPL*1*A*4, *CpSCPL*1*A*5, *CpSCPL*1*A*6, *CpSCPL*1*A*10, and *CpSCPL*1*A*11 shared a close relationship with the highly expressed gene TEA034031 in *C. sinensis* apical buds, while *CpSCPL*1*A*17 was closely correlated to the highly expressed gene TEA034033 in *C. sinensis* apical buds and young leaves (Figure 5). Phylogenetic analysis offers a reliable approach to examining the correlation between sequence similarity and the functional attributes of proteins belonging to the same clade, and the proteins in the same clade often exhibit similar functions (Wani et al. 2021). The results of phylogenetic analysis in this study suggested that these 11 genes have potential significance as key genes correlated with galloylated catechins synthesis, and urther research is needed to determine their tissue expression patterns in *C. perpetua.*

Compared to other *Camellia* species, flowers of Sect. *Chrysantha* exhibits higher levels of flavonoids and their derivatives. These plant-derived flavonoids, including quercetin, daidzein, and catechins, are instrumental in plant growth and development. Flavonoid metabolites are intricately involved in regulating tissue interactions, defense mechanisms and adaptive responses to environmental changes (Dias et al. 2021). Galloylated catechins is at the forefront of flavonoid research, there are many gene families involved (Huang X et al. 2022). Among them, *SCPL*1*A* are key enzymes in synthesizing the galloylated catechins monomers EC, EGC, ECG, and EGCG. Notably, the downregulation of *SCPL* expression has been observed to reduce the contents of these galloylated catechins monomers in *C. sinensis* leaves (Falcone Ferreyra et al. 2012; Yao et al. 2023). Correlation analysis between structural genes involved in metabolic pathways and their respective metabolic products has emerged as a potent strategy for identifying key genes. We utilized Pearson’s correlation analysis to explore the association between galloylated catechins content and *CpSCPL*1*A* gene expression levels (Figure 7A, B). CG and EGCG content were both positively correlated with 8 genes (*CpSCPL*1*A*1, *CpSCPL*1*A*9, *CpSCPL*1*A*12–17), ECG content was positively related with 5 genes (*CpSCPL*1*A*7, *CpSCPL*1*A*9, and *CpSCPL*1*A*15–17), and GCG content was positively associated with 5 genes (*CpSCPL*1*A*1, *CpSCPL*1*A*11–14) (Pearson correlation coefficient >0.9, *p*<0.05, Figure 7B), indicating that gene expression of these 11 *CpSCPL*1*A* genes might be responsible for the uniquely high contents of production of galloylated catechins. Functional experimentation is needed to determine whether some or all of these *SCPL* genes are involved in catechins galloylatedification. In addition, transcription factors can regulate gene expression by binding to DNA (Han et al. 2016; Wang et al. 2018; Xu et al. 2018), which may further regulate the production of galloylated catechins in *C. perpetua* flowers. However, in *C. perpetua* flowers, the interactions between transcription factors, structural genes, and metabolite biosynthesis are complex, so more research is needed to explore their mechanisms of action.

## Conclusions

In this study, we constructed the first full-length transcriptome of *C. perpetua* flowers, resulting in 38.30 Gb of sequencing data and the identification of 57,487 transcripts, including 42,883 simple sequence repeat (SSR) sites, 2,602 lncRNAs and 41,961 protein-coding sequences. Subsequently, 17 members of the *CpSCPL*1*A* gene family were identified, all containing conserved motifs 1, 4, 5, 6 and 8. Subcellular localization predictions suggest that *CpSCPL*1*A* proteins are localized in the vacuole and peroxisome. By the correlation analysis between the gene expression of 17 *CpSCPL*1*A* genes and the content of galloylated catechins at four developmental stages of *C. perpetua* flowers, we identified 11 key genes (*CpSCPL*1*A*1–2, *CpSCPL*1*A*4–6, *CpSCPL*1*A*10–13, *CpSCPL*1*A*17) involved in the biosynthesis of galloylated catechins. The findings of this study expanded the transcriptomic resources available for *C. perpetua* and provided valuable genetic information for the future identification of genes involved in the biosynthesis of galloylated catechins compounds in this species.

## Abbreviations

ANR: anthocyanidin reductase
CDS: coding sequence
CHI: chalcone isomerase
CHS: chalcone synthase
DFR: dihydroflavonol reductase
FLNC: full-length non-chimeric
HMM: hidden Markov model
HPLC: high-performance liquid chromatography
LAR: leucoanthocyanidin reductase
lncRNA: long non-coding RNA
NJ tree: neighbor-joining tree
ONT: Oxford Nanopore Technologies
PCR: polymerase chain reaction
RT-qPCR: reverse transcription-quantitative polymerase chain reaction
*SCPL*1*A*s: type 1A serine carboxypeptidase-like acyltransferases
SSR: simple sequence repeat

## Description of supplementary files

Supplementary Figure S1. The biosynthesis pathway of catechins in *C. perpetua*.

Supplementary Figure S2. The HPLC standard chromatograms of CG, EGCG, ECG and GCG, and the types of compounds detected in the different stages of S1-S4(from top to bottom are t).

Supplementary Table S1. Background genes for HMMER and DIAMOND identification.

Supplementary Table S2. Phylogenetic analysis of *CpSCPL*1*A* proteins.

Supplementary Table S3. Primers for *CpSCPL*1*A* genes.

Supplementary Table S4. Transcripts function annotation in different database.

Supplementary Table S5. Number of key genes and enzymes involved in flavonoid biosynthesis.

Supplementary Table S6. Sequences of the motifs of *CpSCPL*1*A*.

## References

[RAyadi2018] Ayadi L, Motorin Y, Marchand V (2018) Quantification of 2′-O-Me residues in RNA using next-generation sequencing (Illumina RiboMethSeq Protocol). In: Gaspar I (ed) *RNA Detection. Methods in Molecular Biology*. Humana Press, New York, pp 1649 10.1007/978-1-4939-7213-5_2 29130188

[RBaranwal2022] Baranwal A, Aggarwal P, Rai A, Kumar N (2022) Pharmacological actions and underlying mechanisms of catechin: A review. *Mini Rev Med Chem* 22: 821–833 34477517 10.2174/1389557521666210902162120

[RBeier2017] Beier S, Thiel T, Münch T, Scholz U, Mascher M (2017) MISA-web: A web server for microsatellite prediction. *Bioinformatics* 33: 2583–2585 28398459 10.1093/bioinformatics/btx198 PMC5870701

[RBerardini2015] Berardini TZ, Reiser L, Li D, Mezheritsky Y, Muller R, Strait E, Huala E (2015) The Arabidopsis Information Resource: Making and mining the “gold standard” annotated reference plant genome. *Genesis* 53: 474–485 26201819 10.1002/dvg.22877 PMC4545719

[RBuchfink2015] Buchfink B, Xie C, Huson D (2015) Fast and sensitive protein alignment using DIAMNOND. *Nat Methods* 12: 59–60 25402007 10.1038/nmeth.3176

[RCapella2009] Capella-Gutiérrez S, Silla-Martínez JM, Gabaldón T (2009) trimAL: A tool for automated alignment trimming in large-scale phylogenetic analyses. *Bioinformatics* 25: 1972–1973 19505945 10.1093/bioinformatics/btp348 PMC2712344

[RChen2020] Chen C, Chen H, Zhang Y, Thomas HR, Frank MH, He Y, Xia R (2020) Tbtools: An integrative toolkit developed for interactive analyses of big biological data. *Mol Plant* 13: 1194–1202 32585190 10.1016/j.molp.2020.06.009

[RChen2019] Chen X, Li J, Wang X, Zhong L, Tang Y, Zhou X, Liu Y, Zhan R, Zheng H, Chen W, et al. (2019) Full-length transcriptome sequencing and methyl jasmonate-induced expression profile analysis of genes related to patchoulol biosynthesis and regulation in *Pogostemon cablin.* *BMC Plant Biol* 19: 266 31221095 10.1186/s12870-019-1884-x PMC6585090

[RChou2010] Chou KC, Shen H (2010) Plant-mPLoc: A top-down strategy to augment the power for predicting plant protein subcellular localization. *PLoS One* 5: e11335 20596258 10.1371/journal.pone.0011335 PMC2893129

[RDe2023] De Coster W, Rademakers R (2023) NanoPack2: Population-scale evaluation of long-read sequencing data. *Bioinformatics* 39: btad311 37171891 10.1093/bioinformatics/btad311 PMC10196664

[d67e2367] Deléage G (2017) ALIGNSEC: Viewing protein secondary structure predictions within large multiple sequence alignments. *Bioinformatics* 33: 3991–3992 28961944 10.1093/bioinformatics/btx521

[RDias2021] Dias MC, Pinto DCGA, Silva AMS (2021) Plant flavonoids: Chemical characteristics and biological activity. *Molecules* 26: 5377 34500810 10.3390/molecules26175377 PMC8434187

[RFalcone2012] Falcone Ferreyra ML, Rius SP, Casati P (2012) Flavonoids: Biosynthesis, biological functions, and biotechnological applications. *Front Plant Sci* 3: 222 23060891 10.3389/fpls.2012.00222 PMC3460232

[RGao2024] Gao Q, Tong W, Li F, Wang Y, Wu Q, Wan X, Xia E (2024) TPIA2: An updated tea plant information archive for *Camellia* genomics. *Nucleic Acids Res* 52(D1): D1661–D1667 37650644 10.1093/nar/gkad701 PMC10767884

[RGuo2023] Guo J, Wang Y, Li J, Zhang J, Wu Y, Wang G (2023) Overview and recent progress on the biosynthesis and regulation of flavonoids in *Ginkgo biloba* L. *Int J Mol Sci* 24: 14604 37834050 10.3390/ijms241914604 PMC10572177

[RHan2016] Han Y, Wu M, Cao L, Yuan W, Dong M, Wang X, Chen W, Shang F (2016) Characterization of OfWRKY3, a transcription factor that positively regulates the carotenoid cleavage dioxygenase gene OfCCD4 in *Osmanthus fragrans.* *Plant Mol Biol* 91: 485–496 27106478 10.1007/s11103-016-0483-6

[RHuang2023] Huang C, Wen W, Li Q, Wang M, Xiao S, Zhang X, Huang Q, Qian G, Li L, Xu D (2023) Identification, characterization and expression analysis of the 4-coumarate-coa ligase gene family in *Bletilla striata.* *Gene Rep* 32: 101785

[RHuang2022a] Huang F, Li Y, Yang P, Liu Z, Huang J, Xiong L, Li J (2022) Relationship between theanine, catechins and related genes reveals accumulation mechanism during spring and summer in tea plant (*Camellia sinensis* L.). *Sci Hortic (Amsterdam)* 302: 111142

[RHuang2022b] Huang X, Yu S, Chen S, Lin H, Luo Y, Li J, Zhu M, Wang K (2022) Complementary transcriptomic and metabolomics analysis reveal the molecular mechanisms of EGCG3″me biosynthesis in *Camellia sinensis.* *Sci Hortic (Amsterdam)* 304: 111340

[RJain2016] Jain M, Olsen HE, Paten B, Akeson M (2016) The Oxford Nanopore Minion: Delivery of nanopore sequencing to the genomics community. *Genome Biol* 239: 17–29 10.1186/s13059-016-1103-0 PMC5124260 27887629

[RJiang2023] Jiang H, Qin H, Tang J, Chen Z, Zou R, Jiang Y, Xiao W, Chai S (2023) Flowering nectar secretion and pollinators activity of *Camellia perpetua* in different seasons, a perpetual flowering plant in south China. Preprint: https://doi.org/10.22541/au.167785049.94248293/v1

[RJohnson2012] Johnson R, Bryant S, Huntley AL (2012) Green tea and green tea catechin extracts: An overview of the clinical evidence. *Maturitas* 73: 280–287 22986087 10.1016/j.maturitas.2012.08.008

[RKong2007] Kong L, Zhang Y, Ye ZQ, Liu XQ, Zhao SQ, Wei L, Gao G (2007) CPC: Assess the protein-coding potential of transcripts using sequence features and support vector machine. *Nucleic Acids Res* 35(suppl_2): W345–W349 17631615 10.1093/nar/gkm391 PMC1933232

[RKumar2018] Kumar S, Stecher G, Li M, Knyaz C, Tamura K (2018) MEGA X: Molecular evolutionary genetics analysis across computing platforms. *Mol Biol Evol* 35: 1547–1549 29722887 10.1093/molbev/msy096 PMC5967553

[RLetunic2021] Letunic I, Bork P (2021) Interactive Tree Of Life (iTOL) v5: An online tool for phylogenetic tree display and annotation. *Nucleic Acids Res* 49(W1): W293–W296 33885785 10.1093/nar/gkab301 PMC8265157

[RLi2015] Li W, Cowley A, Uludag M, Gur T, McWilliam H, Squizzato S, Park YM, Buso N, Lopez R (2015) The EMBL-EBI bioinformatics web and programmatic tools framework. *Nucleic Acids Res* 43(W1): W580–W584 25845596 10.1093/nar/gkv279 PMC4489272

[RLi2020] Li XM, Li CN, Lu JS, Huang ZW, Cui XQ, Zhang ZB, Zhou JY, Bu ZY (2020) Analysis and evaluation of active ingredient in flowers of 6 species of *Camellia* sect. *Chrysantha* Chang. *Food Res and Dev* 41: 33–37 (in Chinese)

[RLiu2018] Liu L, Wu Y, Liao Z, Xiong J, Wu F, Xu J, Lan H, Tang Q, Zhou S, Liu Y, et al. (2018) Evolutionary conservation and functional divergence of the *LFK* gene family play important roles in the photoperiodic flowering pathway of land plants. *Heredity (Edinb)* 120: 310–328 29225355 10.1038/s41437-017-0006-5 PMC5842218

[RLivak2001] Livak KJ, Schmittgen TD (2001) Analysis of relative gene expression data using real-time quantitative PCR and the 2^−ΔΔct^ method. *Methods* 25: 402–408 11846609 10.1006/meth.2001.1262

[RLu2016] Lu H, Giordano F, Ning Z (2016) Oxford Nanopore MinION sequencing and genome assembly. *Genomics Proteomics Bioinformatics* 14: 265–279 27646134 10.1016/j.gpb.2016.05.004 PMC5093776

[RMestre2023] Mestre-Tomás J, Liu T, Pardo-Palacios F, Conesa A (2023) SQANTI-SIMq: A simulator of controlled transcript novelty for lrRNA-seq benchmark. *Genome Biol* 24: 286 38082294 10.1186/s13059-023-03127-0 PMC10712166

[RNi2021] Ni L, Wang Z, Liu X, Wu S, Hua J, Yin Y, Li H, Gu C (2021) Transcriptome analysis of salt stress in *Hibiscus hamabo* Sieb. et Zucc based on Pacbio full-length transcriptome sequencing. *Int J Mol Sci* 23: 138 35008561 10.3390/ijms23010138 PMC8745204

[RPark2018] Park E, Pan Z, Zhang Z, Lin L, Xing Y (2018) The expanding landscape of alternative splicing variation in human populations. *Am J Hum Genet* 102: 11–26 29304370 10.1016/j.ajhg.2017.11.002 PMC5777382

[RPeng2011] Peng X, Yu D, Fen B, Tang L, Wang Y, Shi L (2011) Chemical constituents from the flowers of *Camellia nitidissima*. *Guihaia* 31: 550–553, 568

[RPetersen2019] Petersen LM, Martin IW, Moschetti WE, Kershaw CM, Tsongalis GJ (2019) Third-generation sequencing in the clinical laboratory: Exploring the advantages and challenges of nanopore sequencing. *J Clin Microbiol* 58: e01315–e01319 31619531 10.1128/JCM.01315-19 PMC6935936

[RPotter2018] Potter SC, Luciani A, Eddy SR, Park Y, Lopez R, Finn RD (2018) HMMER web server: 2018 update. *Nucleic Acids Res* 46(W1): W200–W204 29905871 10.1093/nar/gky448 PMC6030962

[RPunyasiri2004] Punyasiri PAN, Abeysinghe ISB, Kumar V, Treutter D, Duy D, Gosch C, Martens S, Forkmann G, Fischer TC (2004) Flavonoid biosynthesis in the tea plant *Camellia sinensis*: Properties of enzymes of the prominent epicatechin and catechin pathways. *Arch Biochem Biophys* 431: 22–30 15464723 10.1016/j.abb.2004.08.003

[RQi2016] Qi J (2016) Isolation, identification and activity evaluation of chemical constituents from *Camellia nitidissima*. Ph.D.Thesis, *Nanjing University of Science and Technology*, Beijing, China

[RRani2012] Rani A, Singh K, Ahuja PS, Kumar S (2012) Molecular regulation of catechins biosynthesis in tea [*Camellia sinensis* (L.) O. Kuntze]. *Gene* 495: 205–210 22226811 10.1016/j.gene.2011.12.029

[RRice2000] Rice P, Longden I, Bleasby A (2000) EMBOSS: The European molecular biology open software suite. *Trends Genet* 16: 276–277 10827456 10.1016/s0168-9525(00)02024-2

[RRozewicki2019] Rozewicki J, Li S, Amada KM, Standley DM, Katoh K (2019) MAFFT-DASH: Integrated protein sequence and structural alignment. *Nucleic Acids Res* 47(W1): W5–W10 31062021 10.1093/nar/gkz342 PMC6602451

[RSahlin2021] Sahlin K, Medvedev P (2021) Error correction enables use of Oxford Nanopore Technology for reference-free transcriptome analysis. *Nat Commun* 12: 2 33397972 10.1038/s41467-020-20340-8 PMC7782715

[RSatam2023] Satam H, Joshi K, Mangrolia U, Waghoo S, Zaidi G, Rawool S, Thakare RP, Banday S, Mishra AK, Das G, et al. (2023) Next-generation sequencing technology: Current trends and advancements. *Biology (Basel)* 12: 997 37508427 10.3390/biology12070997 PMC10376292

[RSigurpalsdottir2024] Sigurpalsdottir BD, Stefansson OA, Holley G, Beyter D, Zink F, Hardarson MÞ, Sverrisson SÞ, Kristinsdottir N, Magnusdottir DN, Magnusson OÞ, et al. (2024) A comparison of methods for detecting DNA methylation from long-read sequencing of human genomes. *Genome Biol* 25: 69 38468278 10.1186/s13059-024-03207-9 PMC10929077

[RSinija2008] Sinija VR, Mishra HN (2008) Green tea: Health benefits. *J Nutr Environ Med* 17: 232–242

[RSwarbreck2008] Swarbreck D, Wilks C, Lamesch P, Berardini TZ, Garcia-Hernandez M, Foerster H, Li D, Meyer T, Muller R, Ploetz L, et al. (2008) The Arabidopsis Information Resource (TAIR): Gene structure and function annotation. *Nucleic Acids Res* 36(Database): D1009–D1014 17986450 10.1093/nar/gkm965 PMC2238962

[RSzklarczyk2023] Szklarczyk D, Kirsch R, Koutrouli M, Nastou K, Mehryary F, Hachilif R, Gable AL, Fang T, Doncheva NT, Pyysalo S, et al. (2023) The STRING database in 2023: Protein–protein association networks and functional enrichment analyses for any sequenced genome of interest. *Nucleic Acids Res* 51(D1): D638–D646 36370105 10.1093/nar/gkac1000 PMC9825434

[RTang2009] Tang Q, Luo Y, Huang L, Lu M, Tang L, Shi L, Feng B, Wang Y (2009) Determination of chemical constituents in section *Chrysantha* chang. *Lishizhen Med Mater Med Res* 20: 769–771

[RTang2024] Tang X (2024) A latent hidden Markov model for process data. *Psychometrika* 89: 205–240 37934358 10.1007/s11336-023-09938-1

[RWang2018] Wang P, Yang C, Chen H, Luo L, Leng Q, Li S, Han Z, Li X, Song C, Zhang X, et al. (2018) Exploring transcription factors reveals crucial members and regulatory networks involved in different abiotic stresses in *Brassica napus* L. *BMC Plant Biol* 18: 202 30231862 10.1186/s12870-018-1417-z PMC6146658

[RWani2021] Wani SH, Anand S, Singh B, Bohra A, Joshi R (2021) Wrky transcription factors and plant defense responses: Latest discoveries and future prospects. *Plant Cell Rep* 40: 1071–1085 33860345 10.1007/s00299-021-02691-8

[RWei2018] Wei C, Yang H, Wang S, Zhao J, Liu C, Gao L, Xia E, Lu Y, Tai Y, She G, et al. (2018) Draft genome sequence of *Camellia sinensis* var. *sinensis* provides insights into the evolution of the tea genome and tea quality. *Proc Natl Acad Sci USA* 115: E4151–E4158 29678829 10.1073/pnas.1719622115 PMC5939082

[RWu2020] Wu L, Zheng H, Chen W, Li W, Chen D, Ye W (2020) Performance and thinking on introduction and cultivation of *Camellia* sect. *Chrysantha* chang in Fujian. *Fujian Forestry Sci and Tech* 47: 109–115

[RXiong2013] Xiong L, Li J, Li Y, Yuan L, Liu S, Huang JA, Liu Z (2013) Dynamic changes in catechin levels and catechin biosynthesis-related gene expression in albino tea plants (*Camellia sinensis* L.). *Plant Physiol Biochem* 71: 132–143 23911731 10.1016/j.plaphy.2013.06.019

[RXu2018] Xu FC, Liu HL, Xu YY, Zhao JR, Guo YW, Long L, Gao W, Song CP (2018) Heterogeneous expression of the cotton R2R3-MYB transcription factor GbMYB60 increases salt sensitivity in transgenic *Arabidopsis.* *Plant Cell Tissue Organ Cult* 133: 15–25

[RYao2023] Yao X, Qi Y, Chen H, Zhang B, Chen Z, Lu L (2023) Comparative transcriptomic and proteomic analysis of nutritional quality-related molecular mechanisms of ‘Qianmei 419’ and ‘Qianfu 4’ varieties of *Camellia sinensis.* *Gene* 865: 147329 36870427 10.1016/j.gene.2023.147329

[RYu2021] Yu H, Liu M, Yin M, Shan T, Peng H, Wang J, Chang X, Peng D, Zha L, Gui S (2021) Transcriptome analysis identifies putative genes involved in triterpenoid biosynthesis in *Platycodon grandiflorus.* *Planta* 254: 34 34291354 10.1007/s00425-021-03677-2

[RYu2023] Yu JJ, Cui J, Huang H, Cen DC, Liu F, Xu ZF, Wang Y (2023) Identification of flowering genes in *Camellia perpetua* by comparative transcriptome analysis. *Funct Integr Genomics* 24: 2 38066213 10.1007/s10142-023-01267-x

