# Supplementary material for: Full-length transcriptome of Camellia perpetua reveals candidate SCPL1A gene family members involved in galloylated catechins biosynthesis
Source: Plant Biotechnol (Tokyo). 2025 Dec 25;42(4):389–400. doi: 10.5511/plantbiotechnology.25.0317a (PMC12781911; doi:10.5511/plantbiotechnology.25.0317a)
Supplement: Supplementary Data [file plantbiotechnology-42-4-25.0317a-s001.pdf]

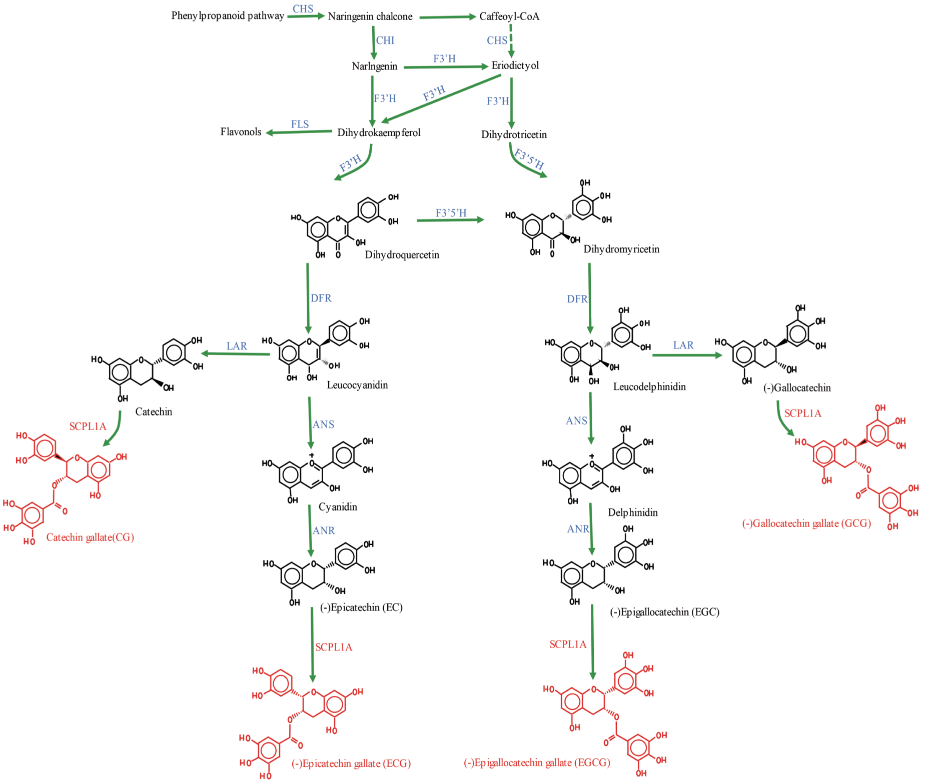

Supplementary Figure S1. The biosynthesis pathway of catechins in *C. perpetua*.

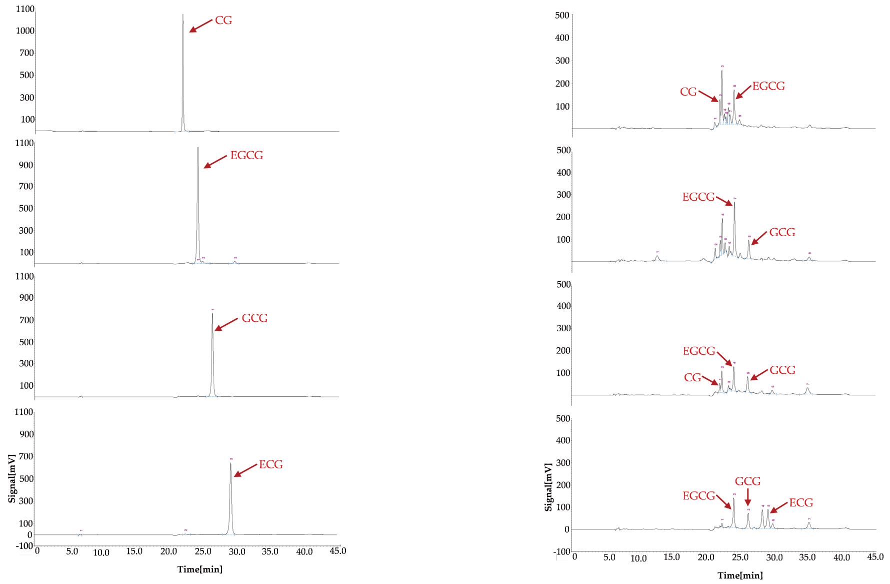

A B

Supplementary Figure S2. A: The HPLC standard chromatograms of CG, EGCG, ECG and GCG. B: The types of compounds detected in the different stages of S1-S4(from top to bottom are t).

Supplementary Table S1. Background Genes for HMMER and DIAMOND Identification.

Camellia sinensis	*Arabidopsis thaliana*	*Zea mays*
TEA034031.1	AT1G33540.1	BFI04783
TEA034032.1	AT1G73270.1	BFI08293
TEA020540.1	AT1G73280.1	BFI10053
TEA010715.1	AT1G73290.1	BFI11663
TEA034056.1	AT1G73300.1	BFI13136
TEA009664.1	AT1G73310.1	BFI14846
TEA016469.1	AT2G22920.2	BFI19822
TEA016463.1	AT2G22970.3	BFI20565
TEA034017.1	AT2G22980.4	BFI24987
TEA034055.1	AT2G22990.3	BFI28520
TEA034033.1	AT2G23000.1	BFI30277
TEA034034.1	AT2G23010.2	BFI31886
TEA034036.1	AT3G10450.1	BFI33383
TEA023444.1	AT3G12203.1	BFI35175
TEA034039.1	AT3G12220.1	BFI40193
TEA023451.1	AT3G12230.1	BFI40936
TEA023432.1	AT3G12240.1	
TEA027270.1	AT5G09640.1	
TEA034028.1	AT5G36180.1	
TEA034050.1		
TEA034049.1		
TEA000223.1		

Supplementary Table S2. Phylogenetic analysis of *CpSCPL1A* proteins

Camellia sinensis	*Camellia perpetua*
TEA034031.1	*CpSCPL1A*1
TEA034032.1	*CpSCPL1A*2
TEA020540.1	*CpSCPL1A*3
TEA010715.1	*CpSCPL1A*4
TEA034056.1	*CpSCPL1A*5
TEA009664.1	*CpSCPL1A*6
TEA016469.1	*CpSCPL1A*7
TEA016463.1	*CpSCPL1A*8
TEA034017.1	*CpSCPL1A*9
TEA034055.1	*CpSCPL1A*10
TEA034033.1	*CpSCPL1A*11
TEA034034.1	*CpSCPL1A*12
TEA034036.1	*CpSCPL1A*13
TEA023444.1	*CpSCPL1A*14
TEA034039.1	*CpSCPL1A*15
TEA023451.1	*CpSCPL1A*16
TEA023432.1	*CpSCPL1A*17
TEA027270.1	
TEA034028.1	
TEA034050.1	
TEA034049.1	
TEA000223.1	

Supplementary Table S3. Primers for *CpSCPL1A* genes

Primer Name	Forward Primer (5' to 3')	Reverse Primer(5' to 3')
CpSCPL1A1	AGAATATTGGGACTTCCTGG	TAAGTTTAATCCCGAGGCTG
CpSCPL1A2	TGGTGAATAAGGCCTGTTTG	TGAGTGAACTCGACATTCGG
CpSCPL1A3	AAGATGCACTTGTCTCCCAG	GAGATGTAGTTTGGGAAGGC
CpSCPL1A4	TTTCATCCTAGCTGCAGTTG	GGGAAGAAAGGACCTAACTC
CpSCPL1A5	GAGGTTTATCCACAAGAGGG	CTCTTGCATTGCTCCATAGG
CpSCPL1A6	AAGAACTCTGCGATCGACAC	CTCTTGCATTGCTCCATAGG
CpSCPL1A7	GGGAGGGCACTTTTCTATTG	TCCACAAACAGCATATTGGC
CpSCPL1A8	CAAGGACCCAATCAACATCG	GGTCCTCCAGTCAACCAAATG
CpSCPL1A9	GATCCCAATGATGTCTCTGC	GCCCTCCAGTCAACCAAATAAC
CpSCPL1A10	TCACACAGTTACCTGGGCAG	TAAAGGGTCTTCCCATCAGG
CpSCPL1A11	GAGGCCATTCGACATGTTGG	ACTATGGACGCGGAATGGTC
CpSCPL1A12	TGCTTGTTCAAACCTGGTAC	GCAAACTTCCATCATTCTGC
CpSCPL1A13	GCAGCACAAGCATTGTGTC	TGGGAGGTAACCAGGAAAAC
CpSCPL1A14	ACATTCCACTCTTTTTTCGC	AATTCGAAATATACCGGACC
CpSCPL1A15	CTCAGCACAAGCTGTCGTAG	CTGTGAGCCAAAGGAAAATG
CpSCPL1A16	GACTGGCCAATTACCATCTC	TCCTGAATACGTATCCCCAC
CpSCPL1A17	GCAGCCAAACTGTTAAGACC	GTTGGCAGTCTGTGTCCATG

Supplementary Table S4. Transcripts Function Annotation in different database

Anno Database	Annotated Number	Annotated Percent (%)
COG	10,753	26.8
GO	29,508	73.7
KEGG	24,593	61.4
KOG	18,948	47.3
Pfam	27,170	67.8
Swiss-Prot	25,092	62.6
eggNOG	2,361	5.9
NR	36,444	91
All Annotated	36,516	91.1

Supplementary Table S5. Number of key genes and enzymes involved in galloylated catechins biosynthesis.

Gene name	Number of transcripts
F3'H	34
DFR	32
F3H	27
LAR	26
SCPL1A	17
CHI	6
CHS	5

Supplementary Table S6. Sequences of the motifs of *CpSCPL1A*.

Motif	Motif Consensus
Motif1	DKRTASDSYVFLLNWLERFPZFKTNDFYIGGESYAGHYVPQLAQTILDGN
Motif2	LTANGLRVWVFSGDHDGRVPVTGTRYSINKLKLHJKTPWRPWYVNGQVGG
Motif3	SZRNPKSDPLLLWLNGGPGCS
Motif4	TLYLNPYSWNKAANILFLDSPVGVGFSYS
Motif5	INLKGFIIGNAVTBDEIDYKGMIDYAHSHALIS
Motif6	FATVRGAGHTVPEYKPKEALSLIDRWLAG
Motif7	DPCSDYYVYAYLNRPDVQEALHANVTKJSYDWEPCSEVIRH
Motif8	QPNVPFTQYSGYVTVNESAGRALFYYFVE
Motif9	DQTSYEIQKYCDFSPNATTQSNECDTATTEA
Motif10	IABIDIYNIYEPLCTSASPTP
